# Families, Schools, and the Longitudinal Changes in Psychological Distress among College Students during the First Year of the COVID-19 Pandemic: Evidence from a National Panel Survey in China

**DOI:** 10.3390/ijerph191912882

**Published:** 2022-10-08

**Authors:** Fengxia Zhu, Yueyun Zhang, Qi Li, Yuanyao Xu, Baozhong Liu

**Affiliations:** 1 Department of Sociology, School of Philosophy and Social Development, Shandong University, Jinan 250100, China; 2 Department of Sociology, School of Humanities, Social Sciences & Law, Harbin Institute of Technology, Harbin 150001, China; 3 Institute of Sociology, Chinese Academy of Social Science, Beijing 100732, China

**Keywords:** COVID-19, psychological distress, socioeconomic status, education background, college students, longitudinal study

## Abstract

Background: The psychological well-being of students in higher education has become an increasingly important concern in the context of the COVID-19 pandemic. The majority of prior studies were cross-sectional and thereby failed to capture the individual changes in mental health over time. Moreover, few studies have explored whether and how socioeconomic and education backgrounds could be related to college students’ mental health changes. This study aimed to fill these gaps. Methods: Data were from a nationwide, two-wave panel survey of college students in China. Baseline information was collected in November 2019, i.e., before the outbreak of the COVID-19 epidemic in China. A follow-up survey was conducted in November and December 2020, when the epidemic was effectively contained in mainland China. In both waves, mental health was assessed with the 10-item Kessler Psychological Distress Scale (K10). The between-wave changes in psychological distress were measured and categorized. Results: Overall, 13.5% of students experienced at least a one-standard-deviation increase in psychological distress over the one-year follow-up. Results from both bivariate and multivariable analysis showed that this marked distress increase was more pronounced among vocational college students (vs. academic undergraduate and postgraduate students) and those with lower levels of academic performance. In contrast, family socioeconomic status (as measured by parental education, family economic condition, and current residence) was not associated with distress changes over time. Conclusions: These findings highlight the importance of the educational disparities within the higher education system in understanding the mental health dynamics of college students in the context of the COVID-19 pandemic.

## 1. Introduction

The coronavirus disease 2019 (COVID-19), since its initial outbreak in late 2019, has been rapidly spreading across the globe for almost three years. As of 18 August 2022, the World Health Organization reported over 590 million confirmed cases, including 6.4 million deaths globally since the pandemic [1]. Apart from its direct influences on physical health, the COVID-19 pandemic has been increasingly found to have profound psychological implications. As an expanding group among young adults, students in higher education have received particular attention in terms of their psychological well-being with the progress of the COVID-19 pandemic [2,3,4,5]. 

Students in higher education are generally at the formative stage of the life course or emerging adulthood, and thus, they might be more vulnerable to mental health problems compared with other age groups [6]. In the context of the COVID-19 pandemic, many studies have paid attention to the prevalence of various psychological symptoms among college students from different countries and cultures [7,8,9], and a growing body of research has explored the risk or protective factors associated with the mental health of college students [10,11]. This study aimed to contribute to these discussions with empirical evidence from China. We utilized data from a national panel survey that had collected baseline information just before the outbreak of the COVID-19 pandemic and follow-up information one year later. We asked two specific questions: (1) whether and to what extent the psychological distress of college students had changed over a one-year follow-up period and (2) whether and how the distress change over time could be related to students’ family socioeconomic status and education background. 

Before proceeding to the data analysis, let us provide a broad sketch of the prior literature that has motivated our study, First, given the persistence of the pandemic, a growing body of literature has devoted attention to the temporal changes in college students’ mental health, with evidence from China [9,12], the United States [8], Switzerland [7,10], and Bengal [13,14,15]. However, the empirical findings yielded from these studies were inconsistent. Some studies found that college students’ mental health worsened in the few months following the pandemic outbreak [5,16,17]. Some other evidence suggested that in the long run, their mental health was quite resilient [2,18,19], or they just experienced minor changes during the COVID-19 pandemic [4,20]. It is also worthwhile to note that there was always a particular group of notably vulnerable students suffering from persistent [5,21] or even deteriorating [12,22] mental health problems. These inconsistent findings taken together highlighted the necessity of maintaining a constant focus on the mental health dynamics of college students across time and space.

Second, much attention has also been devoted to the factors influencing college students’ psychological well-being. The factors that received much scrutiny include individual-level demographic characteristics such as gender and age [23,24] and macro-level institutional factors related to administrative efforts such as isolation policies and social support [23,25,26]. Few further attempts, however, have been made to explore the associations of socioeconomic status and education background of students in higher education with their psychological well-being. On the one hand, it is indeed well known that family socioeconomic status has always been one of the crucial factors affecting the mental health of youth and adolescents [27,28,29]. A few recent studies discussed the role of family background in students’ mental health during the COVID-19 pandemic but yielded conflicting results. While some studies showed that the proportion of students with mental health problems gradually decreased along with increasing family income [26,30], some other studies indicated that family background factors did not play a role in the discrepancies in college students’ mental health [14,31]. On the other hand, the education differentiation among college students has gained increasing prominence with the expansion of the higher education system worldwide [32]. Indeed, quite a few studies have linked the disparities in academic major, grade level, and school type within the higher education system to various educational and economic consequences [33,34,35] and, most notably, to the mental health discrepancies among students in the higher education system [36,37,38]. Against the backdrop of the COVID-19 pandemic, only a handful of studies have examined the association of education differences with the mental health of college students, and the results were far from conclusive [30,39].

Taken together, despite the persistence of the COVID-19 pandemic and the increasing awareness of the psychological vulnerability of higher education students across countries and contexts, the existing research is still limited in capturing individual changes in mental health over time, primarily due to the lack of appropriate data. Moreover, few attempts have been made to systematically investigate whether and how the longitudinal changes in mental health can be associated with students’ family socioeconomic status and education background. Empirical answers to these questions can be of considerable interest to scholars and policymakers concerned with the long-term psychological well-being of college students in the context of the COVID-19 pandemic. Furthermore, the findings concerning the influential factors involving family socioeconomic status and education background may help the higher education sector design and implement tailored strategies to mitigate the psychological problems of college students.

This study represents one of the first attempts to track the longitudinal changes in mental health and their potential correlates regarding college students’ family socioeconomic status and education background. We used data from a nationwide, two-wave panel survey of Chinese higher-education students. The baseline information was collected before confirmed cases of COVID-19 were reported in China, and the follow-up survey was conducted in October 2020, when there had been no new confirmed cases in mainland China for nearly two months in a row [40]. It is now clear that the two surveys were completed just before and after the peak of the COVID-19 pandemic, thereby allowing us to capture the changes in college students’ psychological distress during the COVID-19 pandemic.

Before we proceed to the method section, it is helpful to better situate our study in the Chinese context of the first year of the pandemic. When the baseline survey was conducted in November 2019, Chinese college students were carrying out their normal school life. In January 2020, Chinese experts officially declared that the newly detected coronavirus could be transmitted from person to person when most college students in China were spending their winter vacation at home. In March, the World Health Organization declared COVID-19 a pandemic when the spring semester was gradually starting in China. In the next few months, the epidemic had been effectively contained, but students were not allowed to return to campus and were required to take their registered courses via the internet [41]. When it came to the fall semester in 2020, students in most colleges were organized to return to school under refined guidelines [42]. In theory, they could attend offline courses, but these courses were often transferred online when newly local confirmed cases were reported. It was against this backdrop that we conducted the follow-up survey in October 2020.

## 2. Materials and Methods

### 2.1. Participants and Procedures

Participants were from a nationwide, two-wave panel survey conducted at two time points: before and after the peak of the COVID-19 pandemic in China. A stratified, multistage sampling framework was applied in the survey. First, all the provincial regions in China were divided into seven strata, including the North, the East, the South, the Central, the Northeast, the Northwest, and the southwest. Within each stratum, two or three colleges were chosen. Additionally, colleges from the region of east China were oversampled because of the high concentration of higher institutions there. The survey included colleges of different types and ranks. As a result, 20 colleges were obtained to form the primary sampling units (PSUs). Second, eight majors were randomly sampled within each selected college, with assistance from college staff. Third, within each selected major, one class was randomly sampled from each grade. Finally, all the students in the sampled classes were invited to participate in the survey. Our baseline survey was conducted just before the outbreak of the COVID-19 pandemic, with an independent aim of collecting information about college students’ academic activities and social values as well as health conditions. With technical assistance from each college, respondents were asked to finish a self-administered questionnaire embedded in a smartphone application developed specifically for the study. A student respondent could finish the questionnaire on campus or at home. Each respondent could only use his or her unique student ID to log in the system and then gain access to the questionnaire. Further, one digital device could only allow access from one student ID. Thus, multiple submissions from a single respondent were easily identified and blocked.

**Figure 1:**
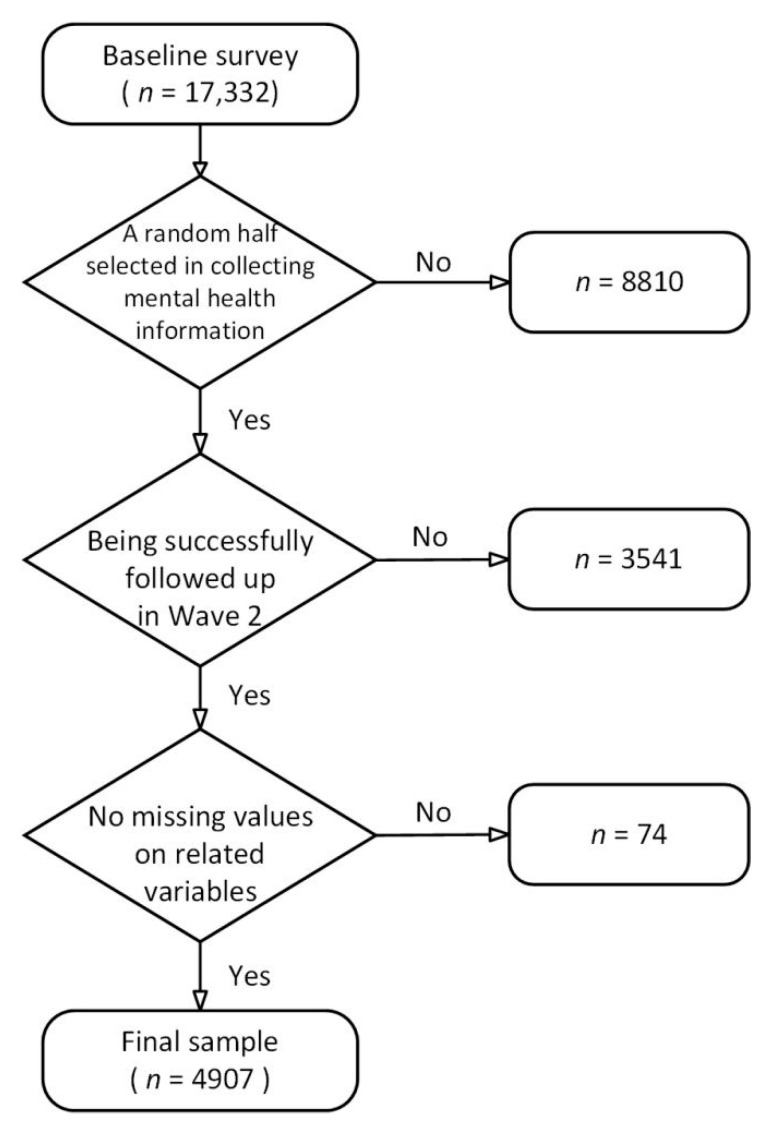
Flowchart for obtaining the final analytical sample.

We obtained 17,332 participants in the pre-COVID-19 survey wave. To save time and space, a random half (*n* = 8522) was selected and assigned to the mental health module in the questionnaire. A comparison of descriptive statistics between the selected random-half sample and the dropped random-half sample is reported in Appendix A Table A1. Of the 8522 respondents who were interviewed at the baseline survey, 3541 students were not followed up in the second wave, due to graduation, dropping out, or other unknown reasons. In addition, those with missing values on related variables in either survey wave (*n* = 74) were excluded. As such, we obtained a final analytical sample of 4907 respondents. A comparison of descriptive statistics between the followed-up sample and the non-followed-up sample is also reported in Appendix A Table A1. The Institutional Review Board at the Chinese Academy of Social Sciences approved the study protocol, and informed consent was obtained from all respondents before commencing the interview. Figure 1 contains a flow chart that visually renders the steps in obtaining our final analytical sample.

### 2.2. Measures 

#### 2.2.1. The Change in Psychological Distress

The outcome of interest was the individual change in psychological distress over time, i.e., before and after the peak of the COVID-19 pandemic in China. In both survey waves, participants completed the 10-item Kessler Psychological Distress Scale (K10) [43]. The K10 was designed to screen nonspecific psychological distress over a 30-day recall period. It asks about the frequencies of experiencing a series of negative emotional states based on a 5-point Likert-type scale ranging from 1 (none of the time) to 5 (all of the time). The total scores thus range from 10 to 50, with higher values signifying more psychological distress. The psychometric properties of the K10 in the Chinese version have been examined among Chinese college students [44]. In our study, the K10 had a Cronbach’s alpha of 0.923 in the baseline survey and 0.951 in the follow-up survey, indicating high internal consistency. 

To measure the individual changes in psychological distress over the follow-up period, we obtained the change score by subtracting the K10 score at Time 1 from the score at Time 2. The change score had a mean of 0.76 and a standard deviation of 7.43. At the risk of oversimplification, we categorized the change score into three general levels: one standard deviation or more above the mean change score (i.e., a marked distress increase), one standard deviation or more below the mean change score (i.e., a marked distress decrease), and other values between the two. A one-standard-deviation threshold has been adopted in previous cross-sectional [45] and longitudinal [46] research to discern adolescents who are substantially different from the majority in psychological dynamics.

#### 2.2.2. Family Background

Family background factors included parental education, family economic condition, and current residence [41]. Parental education was measured by taking the highest level of education reported for either the mother or the father. The original classification included nine schooling stages ranging from “no education” to “postgraduate and above”. To simplify, we collapsed them into two categories: “below college” and “college and above”. Family economic condition was assessed by self-report by asking, “How is the current economic condition of your family?” and included three categories: “below average”, “about average”, and “above average”. Finally, the current residence was classified as an urban–rural dichotomous variable. Those living in rural areas were usually disadvantaged in public goods and social welfare compared with their urban counterparts [28]. 

#### 2.2.3. Education Background

Education background included respondents’ schooling level, academic performance, and academic major. The schooling level divided respondents into vocational students, academic undergraduates, and academic postgraduates. In China, vocational higher education has been designed to have a 3-year technical training, and students enrolled in this track are more employment-oriented with an emphasis on technical knowledge and practical assessment compared with students enrolled in the 4-year academic colleges [47]. In terms of academic performance, we asked: “Where does your current academic performance rank in the same grade and major?” Options included (1) the top 10%, (2) the interval of 11–25%, (3) the interval of 26–50%, (4) the interval of 51–75%, (5) the interval of 76–90%, (6) the bottom 10%, and (7) unclear. To simplify, we classified the students’ performance rankings into four categories: “below average”, “average”, “above average”, and “unclear”. Major was divided into two broad categories: (1) humanities and social sciences and (2) natural sciences.

#### 2.2.4. Other Covariates

A few other variables were typically included in regression models to better clarify the association between socioeconomic status, education background, and psychological outcomes. Specifically, we included such demographic characteristics as gender (1 = female), age and whether the respondent was an only child (=1). We also included the psychological distress status at T1, that is, before the peak of the COVID-19 pandemic, adjusting for the initial mental health status of the respondents.

### 2.3. Statistical Analysis

Firstly, descriptive statistics were reported as percentages and frequencies for categorical variables. Then cross-tabulations with chi-square tests were used to examine the distribution of psychological distress changes across subgroups with different family and education backgrounds. Finally, multivariable regression was used to explore the association between students’ psychological distress changes over time and family background and education background, controlling for demographic characteristics and initial level of psychological distress throughout all analyses. Since our outcome variable was categorical and ordinal, we employed the logit-family models. Odds ratios and 95% confidence intervals were reported. More details are offered where necessary in the results section. Finally, since the respondents were clustered within classes, the significance tests were based on robust standard errors, allowing for correlated residuals within classes [48]. All analyses were performed using Stata for Windows, version 12.0 (StataCorp, College Station, TX, USA).

## 3. Results

Table 1 presents descriptive statistics. Overall, 13.5% of respondents experienced a marked increase in psychological distress during the first year of the COVID-19 pandemic. In terms of demographic characteristics, 56.9% were female, 48.0% were only children, and 14.1% had a parent with a college or above education for family background. Nearly two-thirds of respondents considered their family economic condition average. Urban and rural residents were almost equally divided. As to education background, the sample consisted of 33.5% vocational students, 53.5% academic undergraduates, and 13.0% academic postgraduates. According to official estimates [49], in 2018, vocational students accounted for 37.7% of the whole body of college students, and the gender distribution was 52.5% female and 47.5% male. Overall, the share of vocational students and the share of female students nationwide were quite close to the corresponding figures obtained from our data. Finally, about 60% of the students had intermediate academic performance or above. The respondents studying humanities and social sciences and natural sciences were roughly the same.

Table 2 cross-tabulates psychological distress changes with socioeconomic status and education background. Socioeconomic status was indicated by parental education, family economic condition and current residence. Parental education and current residence were not associated with changes in psychological distress. The relationship between family economic condition and change in psychological distress was not clear, despite its statistical significance. On the one hand, a marked distress decrease occurred the least often among students with above average family economic condition (10.8%). On the other hand, a marked distress increase occurred the least often among students with about average family economic condition (12.0%). Education background was indicated by schooling level, academic performance, and major. The schooling level and academic performance were significantly associated with distress changes. Compared with their counterparts from higher levels of schooling or academic performance, respondents with below-average academic performance (18.4%) or vocational schooling level (15.1%) were more likely to experience a marked increase in psychological distress. It seems that college major did not play a role in shaping the distress changes over time. To offer more background information, we also reported the mean level of psychological distress at the baseline across groups as defined by family and education background in Appendix A Table A2. The results show that family background factors, such as parental education and family income, are associated with baseline distress. Education background, such as schooling level and academic performance, is also associated with baseline distress.

Turning to multivariable analysis, we divided the changes in psychological distress into two broad categories, a marked increase in poor mental health or otherwise, for the sake of simplification. By doing so, we could then employ a binary logistic regression to associate the likelihood of a marked increase in psychological distress with socioeconomic status and education background while controlling for respondents’ demographic factors and their pre-COVID-19 distress collected in the baseline survey. The first two columns of Table 3 show the results, including the odds ratios and 95% confidence intervals. Largely consistent with the bivariate associations illustrated above, we found that students’ schooling level and academic performance were significantly associated with the odds of experiencing a marked increase in psychological distress. For example, the odds of having a marked increase in distress for academic undergraduates were 18% lower (OR = 0.82, 95% CI = 0.67–0.99) than for vocational students, and the odds for academic postgraduates were 20% lower (OR = 0.80, 95% CI = 0.60–0.97) than for vocational students. Moreover, the odds of having a marked distress increase for students with about-average academic performance were 35% lower (OR = 0.65, 95% CI = 0.47–0.88) than for students with below-average performance, and the odds for students with above-average performance were 44% lower (OR = 0.56, 95% CI = 0.41–0.78) than for those with below-average performance. Ceteris paribus, no family socioeconomic factors, including parental education, family economic condition, and current residence, were associated with the likelihood of a marked increase in psychological distress. 

We were aware that dividing the change of psychological distress into two broad categories and then employing binary logistic regression seems to be oversimplified. As a robustness check, we also estimated our models based on the original three-category distress change variable and then fitted an ordered logit regression model. The results are reported in columns 3–4 in Table 3. It can be observed that findings from these models lead to similar conclusions. Therefore, our findings observed from the simple binary logit model were quite robust to alternative model specifications. 

## 4. Discussion

The psychological well-being of college students has been subject to much scrutiny because they are at a critical life stage of developing psychological symptoms. Many lifetime mental disorders have their first onset just prior to or during the typical college years [50]. The most recent studies have documented an increasing prominence of psychological symptoms among adolescents and youth [51] and among college students in particular [16,52,53]. As a result, students in higher education have received particular attention in terms of their psychological well-being with the progress of the COVID-19 pandemic [2,3,4,5]. 

Drawing on data from a nationwide, two-wave panel survey of Chinese college students collected before and after the peak of the COVID-19 pandemic, this study examined the role of college students’ family socioeconomic status and education background in shaping their psychological distress changes during the epidemic. In our sample, the overall distribution of students’ psychological distress was 12.6% marked decrease, 74.0% no marked change, and 13.5% marked increase. We used a threshold of a one-standard deviation in the change score of psychological distress over the one-year follow-up to distinguish between the three conditions. The study added to the literature by presenting the changes in college students’ mental health over the span of the first year of the COVID-19 pandemic. Attention to this change is necessary because compared with physical injury, mental health problems are less perceptible but could profoundly impact college students’ physical and psychological health in the long run [54,55]. 

Moreover, we examined the association between college students’ psychological distress and their family and education background during the COVID-19 pandemic. We employed bivariate and multivariable analysis and obtained consistent findings. Specifically, we found that the marked increase in psychological distress was more pronounced among vocational college students than among academic undergraduate and postgraduate students. In the context of China, vocational students tend to be employment-oriented with an emphasis on technical knowledge and practical assessment [47], and as a result could become more confused about their job prospects in facing the dramatic labor market transformations due to the COVID-19 pandemic. Meanwhile, home quarantine during the pandemic may have indirectly added to vocational students’ psychological distress by keeping them away from practical teaching help [56]. We also found that a marked increase in psychological distress was significantly associated with poor academic performance. Because taking online courses has become a “new normal” [57] for higher education due to the COVID-19 pandemic, college students with lower academic performance may face greater pressure to adapt to the abrupt shifts in the modes of teaching and learning [58]. 

Finally, our results show that family socioeconomic status, as measured by parental education, family economic condition, and current residence, did not play a significant role in shaping the temporal increase in psychological distress among college students, which contrasts with conventional perceptions [27,59] but complements several recent studies from Bangladesh, India, and Canada [14,31,60,61]. Our finding should not be simply interpreted as a refutation of the impact of family background on mental health. As displayed in Appendix A Table A2, family background factors such as parental education and family income were indeed associated with baseline distress. Hence, family background was just not found to be associated with distress change over the follow-up period. It is likely that during the COVID-19 pandemic, almost all college students’ mental health was affected by extensive external disturbances (e.g., home quarantine and school closures) [10,62], which left college students facing unprecedented challenges. Indeed, the COVID-19 pandemic posed a serious threat to the health of every student regardless of their socioeconomic status [7,63]. A recent study depicted that during the COVID-19 pandemic, although urban residence can deliver citizens with better resources, students residing in urban areas still had a significantly higher risk of anxiety than rural students due to the overdensity of the urban population [60]. Taken together, our results suggest that education background may play a more important role than family background in shaping the mental health changes of college students during the COVID-19 pandemic. 

### 4.1. Limitations

Several limitations should also be noted. First, this study may still suffer from the omitted variable bias despite the panel design. For example, students’ social contact, sleep quality, and physical activity during the COVID-19 pandemic have recently been shown to be associated with mental health outcomes [64,65]. Unfortunately, we could not control for these factors due to data limitations. Second, all longitudinal studies have to face participant attrition, which may carry the risk of biased estimation. The current study is no exception, especially in collecting data in the context of the COVID-19 pandemic. In our dataset, the original baseline sample included 17,331 college students, and our final analytical sample included 4907 respondents from the original sample. We had to exclude a random half of the original sample that was not asked to respond to the mental health module in our questionnaire. All demographic characteristics were evenly distributed between the two random-half subsamples, as illustrated in Appendix A Table A1. Appendix A Table A1 further shows that during the follow-up period, those who were not successfully reached showed no significant selection issue regarding family background, education background, or the baseline level of psychological distress. The only exception was major. Therefore, despite a significant shrinkage in sample size, we believe our final sample could still lead to reliable and generalizable findings. Finally, we focused on college students, and thus, the results cannot be generalized to students at lower education levels. The change in psychological well-being among primary and middle school students surely merits further independent examinations.

### 4.2. Implications

Despite the limitations, the findings of this study could aid in the development of intervention strategies aimed at alleviating the mental health problems of college students during the COVID-19 pandemic. First, attention should be paid to the internal heterogeneity among college students in terms of their changes in psychological well-being, and tailored mental health care could be delivered to subgroups whose mental health deteriorated during and after the COVID-19 pandemic. Second, considering the influence of education background on students, the government should give full consideration to the individualized needs of students at different schooling levels and provide them with targeted learning equipment. Meanwhile, colleges and universities should keep a keen eye on the learning difficulties of college students during the epidemic.

## 5. Conclusions

In conclusion, the current study highlights the shifts in psychological status of college students before and after the peak of the COVID-19 pandemic in China, as well as their associations with socioeconomic status and education background. Utilizing data from a nationwide, two-wave panel survey of Chinese college students, we identified a noticeable proportion of college students (13.5%) who experienced at least a one-standard-deviation increase in psychological distress over a one-year follow-up. Moreover, this marked increase in psychological distress was found to be more pronounced among vocational college students (vs. academic undergraduate and postgraduate students) and those with lower levels of academic performance. In contrast, family socioeconomic status (as measured by parental education, family economic condition, and current residence) did not significantly shape temporal increases in psychological distress. These findings could be of help for future studies in different societies that may have faced different present and post-pandemic situations.

## Figures and Tables

**Table 1:** Descriptive statistics (*n* = 4907).

Variables	%	*n*
Change of psychological distress		
A marked decrease	12.6	616
No marked change	74.0	3629
A marked increase	13.5	662
Gender		
Male	43.1	2113
Female	56.9	2794
Age (Mean/SD)	19.8/1.9	4907
Only child		
No	52.1	2554
Yes	48.0	2353
Family economic condition		
Below average	25.5	1253
About average	64.3	3153
Above average	10.2	501
Parental education		
Below college	85.9	4213
College and above	14.1	694
Current residence		
Urban	48.0	2354
Rural	52.0	2553
Schooling level		
Vocational students	33.5	1645
Academic undergraduates	53.5	2626
Academic postgraduates	13.0	636
Academic performance		
Below average	7.2	353
About average	34.5	1695
Above average	25.7	1260
Unclear	32.6	1599
Major		
Humanities and social sciences	48.0	2357
Natural sciences	52.0	2550

Note: A marked increase (decrease) refers to at least a one-standard-deviation above (below) the mean change score.

**Table 2:** Distributions of the individual changes in psychological distress across student groups.

	The Change in Psychological Distress (%)				
	Marked Decrease	No Marked Change	Marked Increase	Total (*n*)	χ^2^
Parental education					3.34
Below college	14.6	72.9	12.6	100.0 (4213)	
College and above	12.1	75.8	12.1	100.0 (694)	
Family economic condition					12.50 *
Below average	16.4	70.6	13.1	100.0 (1253)	
About average	13.9	74.1	12.0	100.0 (3153)	
Above average	10.8	74.9	14.4	100.0 (501)	
Current residence					0.31
Urban	14.1	73.6	12.3	100.0 (2354)	
Rural	14.3	73.0	12.7	100.0 (2553)	
Schooling level					11.25 *
Vocational students	13.1	71.9	15.1	100.0 (1645)	
Academic undergraduates	12.1	75.3	12.6	100.0 (2626)	
Academic postgraduates	13.1	74.1	12.9	100.0 (636)	
Academic performance					16.99 **
Below average	15.9	65.7	18.4	100.0 (353)	
About average	12.7	73.3	13.9	100.0 (1695)	
Above average	12.6	75.2	12.1	100.0 (1260)	
Unclear	11.6	75.4	13.1	100.0 (1599)	
Major					0.72
Humanities & Social Sciences	12.2	74.5	13.4	100.0 (2357)	
Natural Sciences	12.9	73.5	13.6	100.0 (2550)	

Note: A marked increase (decrease) refers to at least a one-standard-deviation above (below) the mean change score. * *p* < 0.05, ** *p* < 0.01.

**Table 3:** Logit models estimating family and education background on individual change in psychological distress over the follow-up period.

	Binary Logit		Ordered Logit	
	OR	95% CI	OR	95% CI
Female	0.71 ***	[0.59, 0.85]	0.81 **	[0.70, 0.94]
Age	0.96	[0.89, 1.03]	0.98	[0.93, 1.04]
Only child	1.17	[0.97, 1.41]	1.11	[0.96, 1.28]
Parental education (College & above = 1)	1.03	[0.78, 1.35]	1.17	[0.95, 1.44]
Family economic condition (Below average = 0)				
About average	0.89	[0.73, 1.09]	0.90	[0.77, 1.06]
Above average	1.03	[0.74, 1.42]	1.04	[0.80, 1.34]
Current residence (Rural = 1)	1.00	[0.82, 1.22]	0.98	[0.84, 1.14]
Schooling level (Vocational students = 0)				
Academic undergraduates	0.82 *	[0.67, 0.99]	0.87 *	[0.83, 0.97]
Academic postgraduates	0.80 *	[0.60, 0.97]	0.84 *	[0.62, 0.96]
Academic performance (Below average = 0)				
About average	0.65 **	[0.47, 0.88]	0.77 *	[0.59, 0.98]
Above average	0.56 ***	[0.41, 0.78]	0.71 *	[0.54, 0.93]
Unclear	0.56 ***	[0.41, 0.78]	0.71 *	[0.55, 0.93]
Major (Natural Sciences = 1)	0.90	[0.76, 1.08]	0.89	[0.77, 1.02]
Psychological distress at T1	0.93 ***	[0.92, 0.94]	0.86 ***	[0.85, 0.87]
*n*	4907		4907	

Note: In the binary logit model, the outcome variable has two values: 1 = a market increase in psychological distress and 0 = otherwise. In the ordered logit model, the outcome variable has three values: 1 = a marked decrease in psychological distress, 2 = no marked change in psychological distress, and 3 = a marked increase in psychological distress. A marked increase (decrease) refers to at least a one-standard-deviation above (below) the mean change score. * *p* < 0.05, ** *p* < 0.01, *** *p* < 0.001. OR—odds ratio; CI—confidence interval.

## Data Availability

Data are available at http://www.pscus.cn/menu2.jsp?langsel=CN (accessed on 1 January 2021), with the permission of the Chinese Academy of Social Sciences.

## Appendix A

**Table A1:** Comparison of descriptive statistics between the baseline random-half sample and the other half and between the followed-up sample and the non-followed-up sample.

	Two Random-Half Subsamples in Baseline Survey			Two Subsamples in the Follow-Up Survey		
Variables	Selected	Non-Selected	*p*-Value	Followed-Up	Non-Followed-Up	*p*-Value
Gender			0.57			<0.01
Male	45.9%	46.3%		43.1%	50.3%	
Female	54.1%	53.7%		56.9%	49.7%	
Age	20.2 (2.0)	20.2 (2.0)	0.74	19.8 (1.9)	20.9 (2.0)	<0.01
Only child			0.41			0.04
No	51.1%	51.8%		52.0%	49.7%	
Yes	48.9%	48.2%		48.0%	50.3%	
Family economic condition			0.57			0.92
Below average	25.6%	25.7%		25.5%	25.8%	
About average	64.1%	64.5%		64.3%	63.8%	
Above average	10.3%	9.8%		10.2%	10.4%	
Parental education			0.30			0.25
Below college	85.5%	84.9%		85.9%	84.9%	
College and above	14.5%	15.1%		14.1%	15.1%	
Current residence			0.49			0.28
Urban	47.5%	47.0%		48.0%	46.8%	
Rural	52.5%	53.0%		52.0%	53.2%	
Schooling level			0.50			0.08
Vocational students	35.5%	35.7%		33.5%	37.6%	
Academic undergraduates	52.2%	52.6%		53.5%	50.2%	
Academic postgraduates	12.3%	11.7%		13.0%	12.2%	
Academic performance			0.23			0.15
Below average	7.3%	7.7%		7.2%	7.5%	
About average	36.1%	34.6%		34.5%	38.6%	
Above average	29.1%	29.6%		25.7%	24.3%	
Unclear	27.5%	28.1%		32.6%	29.6%	
Major			0.79			<0.01
Humanities and Social Sciences	45.1%	44.9%		48.0%	40.5%	
Natural Sciences	54.9%	55.1%		52.0%	59.5%	
Baseline psychological distress (10–50)	19.1 (7.1)	--		18.9 (7.0)	19.5 (7.3)	0.06

Note: Data are presented as mean (SD) for continuous measures. The selected random-half subsample was assigned to the mental health module in the questionnaire. The followed-up subsample was our final sample in the current study, while the non-followed-up subsample was unsuccessfully reached due to graduation, dropping out, or other unknown reasons.

**Table A2:** Baseline level of psychological distress across groups as defined by family and education background.

	Baseline K10 Score		
	Mean	SD	*p*-Value
Parental education			0.04
Below college	19.0	7.1	
College and above	19.7	7.5	
Family economic condition			<0.01
Below average	20.3	7.5	
About average	18.7	6.9	
Above average	18.7	7.1	
Current residence			0.88
Urban	19.1	7.3	
Rural	19.1	6.9	
Schooling level			<0.01
Vocational students	19.0	7.3	
Academic undergraduates	19.5	7.1	
Academic postgraduates	17.8	6.7	
Academic performance			<0.01
Below average	20.9	7.8	
About average	19.3	7.0	
Above average	18.8	7.0	
Unclear	18.8	7.1	
Major			0.99
Humanities & Social Sciences	19.1	7.1	
Natural Sciences	19.1	7.1	

Note: K10 refers to the 10-item Kessler Psychological Distress Scale.

## References

[1] (2022). World Health Organization Coronavirus (COVID-19) Dashboard. https://covid19.who.int/?mapFilter=cases.

[2] Daly M., Robinson E. (2021). Psychological distress and adaptation to the COVID-19 crisis in the United States. J. Psychiatr. Res..

[3] Gonzales G., Loret de Mola E., Gavulic K.A., McKay T., Purcell C. (2020). Mental Health Needs Among Lesbian, Gay, Bisexual, and Transgender College Students During the COVID-19 Pandemic. J. Adolesc. Health.

[4] Pieh C., Budimir S., Humer E., Probst T. (2021). Comparing Mental Health During the COVID-19 Lockdown and 6 Months After the Lockdown in Austria: A Longitudinal Study. Front. Psychiatry.

[5] Von Keyserlingk L., Yamaguchi-Pedroza K., Arum R., Eccles J.S. (2022). Stress of university students before and after campus closure in response to COVID-19. J. Community Psychol..

[6] Lei X.-Y., Xiao L.-M., Liu Y.-N., Li Y.-M. (2016). Prevalence of Depression among Chinese University Students: A Meta-Analysis. PLoS ONE.

[7] Amendola S., von Wyl A., Volken T., Zysset A., Huber M., Dratva J. (2021). A Longitudinal Study on Generalized Anxiety Among University Students During the First Wave of the COVID-19 Pandemic in Switzerland. Front. Psychol..

[8] Frazier P., Liu Y., Asplund A., Meredith L., Nguyen-Feng V.N. (2021). US college student mental health and COVID-19: Comparing pre-pandemic and pandemic timepoints. J. Am. Coll. Health.

[9] Wang C., Pan R., Wan X., Tan Y., Xu L., McIntyre R.S., Choo F.N., Tran B., Ho R., Sharma V.K. (2020). A longitudinal study on the mental health of general population during the COVID-19 epidemic in China. Brain Behav. Immun..

[10] Elmer T., Mepham K., Stadtfeld C. (2020). Students under lockdown: Comparisons of students’ social networks and mental health before and during the COVID-19 crisis in Switzerland. PLoS ONE.

[11] Xu Y., Su S., Jiang Z., Guo S., Lu Q., Liu L., Zhao Y., Wu P., Que J., Shi L. (2021). Prevalence and risk factors of mental health symptoms and suicidal behavior among university students in Wuhan, China during the COVID-19 pandemic. Front. Psychiatry.

[12] Li Y., Zhao J., Ma Z., McReynolds L.S., Lin D., Chen Z., Wang T., Wang D., Zhang Y., Zhang J. (2021). Mental Health Among College Students During the COVID-19 Pandemic in China: A 2-Wave Longitudinal Survey. J. Affect. Disord..

[13] Faisal R.A., Jobe M.C., Ahmed O., Sharker T. (2022). Mental Health Status, Anxiety, and Depression Levels of Bangladeshi University Students During the COVID-19 Pandemic. Int. J. Ment. Health Addict..

[14] Islam M.S., Sujan M.S.H., Tasnim R., Sikder M.T., Potenza M.N., van Os J. (2020). Psychological responses during the COVID-19 outbreak among university students in Bangladesh. PLoS ONE.

[15] Khan A.H., Sultana M.S., Hossain S., Hasan M.T., Ahmed H.U., Sikder M.T. (2020). The impact of COVID-19 pandemic on mental health & wellbeing among home-quarantined Bangladeshi students: A cross-sectional pilot study. J. Affect. Disord..

[16] Lipson S.K., Zhou S., Abelson S., Heinze J., Jirsa M., Morigney J., Patterson A., Singh M., Eisenberg D. (2022). Trends in college student mental health and help-seeking by race/ethnicity: Findings from the national healthy minds study, 2013–2021. J. Affect. Disord..

[17] Shiratori Y., Ogawa T., Ota M., Sodeyama N., Sakamoto T., Arai T., Tachikawa H. (2022). A longitudinal comparison of college student mental health under the COVID-19 self-restraint policy in Japan. J. Affect. Disord. Rep..

[18] Hong W., Liu R.D., Ding Y., Fu X., Zhen R., Sheng X. (2021). Social Media Exposure and College Students’ Mental Health During the Outbreak of COVID-19: The Mediating Role of Rumination and the Moderating Role of Mindfulness. Cyberpsychol. Behav. Soc. Netw..

[19] Zhou J., Zhang Q. (2021). A Survey Study on U.S. College Students’ Learning Experience in COVID-19. Educ. Sci..

[20] Stamatis C.A., Broos H.C., Hudiburgh S.E., Dale S.K., Timpano K.R. (2022). A longitudinal investigation of COVID-19 pandemic experiences and mental health among university students. Br. J. Clin. Psychol..

[21] Wang D., Zhao J., Zhai S., Huang S., Yang Z., Pan Y., Liu X., Fan F. (2022). Longitudinal trajectories of insomnia symptoms among college students during the COVID-19 lockdown in China. J. Psychosom. Res..

[22] Gong S., Li L.Z., Wang S. (2021). Youth mental health before and after the control of the coronavirus disease 2019: A nationally representative cohort study of Chinese college students. J. Affect. Disord. Rep..

[23] Chen R.-N., Liang S.-W., Peng Y., Li X.-G., Chen J.-B., Tang S.-Y., Zhao J.-B. (2020). Mental health status and change in living rhythms among college students in China during the COVID-19 pandemic: A large-scale survey. J. Psychosom. Res..

[24] Prowse R., Sherratt F., Abizaid A., Gabrys R.L., Hellemans K.G.C., Patterson Z.R., McQuaid R.J. (2021). Coping With the COVID-19 Pandemic: Examining Gender Differences in Stress and Mental Health Among University Students. Front. Psychiatry.

[25] Cao W., Fang Z., Hou G., Han M., Xu X., Dong J., Zheng J. (2020). The psychological impact of the COVID-19 epidemic on college students in China. Psychiatry Res..

[26] Fu W., Yan S., Zong Q., Anderson-Luxford D., Song X., Lv Z., Lv C. (2021). Mental health of college students during the COVID-19 epidemic in China. J. Affect. Disord..

[27] Mamun M.A., Hossain M.S., Griffiths M.D. (2019). Mental Health Problems and Associated Predictors Among Bangladeshi Students. Int. J. Ment. Health Addict..

[28] Sun X., Wang Z.J., Li Y.Y., Chan K.Q., Miao X.Y., Zhao S., Wu Y.Q., Li Z., Wu B.M. (2022). Trends of college students’ mental health from 2005 to 2019 and its rural-urban disparities in China. J. Affect. Disord.

[29] Tam C.C., Ye Z., Wang Y., Li X., Lin D. (2021). Self-care behaviors, drinking, and smoking to cope with psychological distress during COVID-19 among Chinese college students: The role of resilience. Psychol. Health.

[30] Rudenstine S., McNeal K., Schulder T., Ettman C.K., Hernandez M., Gvozdieva K., Galea S. (2021). Depression and Anxiety During the COVID-19 Pandemic in an Urban, Low-Income Public University Sample. J. Trauma. Stress.

[31] Howard A.L., Carnrite K.D., Barker E.T. (2022). First-Year University Students’ Mental Health Trajectories Were Disrupted at the Onset of COVID-19, but Disruptions Were Not Linked to Housing and Financial Vulnerabilities: A Registered Report. Emerg. Adulthood.

[32] Gerber T.P., Cheung S.Y. (2008). Horizontal stratification in postsecondary education: Forms, explanations, and implications. Annu. Rev. Sociol..

[33] Hu A., Vargas N. (2015). Economic consequences of horizontal stratification in postsecondary education: Evidence from urban China. High. Educ..

[34] Marginson S. (2016). The worldwide trend to high participation higher education: Dynamics of social stratification in inclusive systems. High. Educ..

[35] Triventi M. (2013). The role of higher education stratification in the reproduction of social inequality in the labor market. Res. Soc. Stratif. Mobil..

[36] Beiter R., Nash R., McCrady M., Rhoades D., Linscomb M., Clarahan M., Sammut S. (2015). The prevalence and correlates of depression, anxiety, and stress in a sample of college students. J. Affect. Disord..

[37] Yang F., Meng H., Chen H., Xu X.H., Liu Z., Luo A., Feng Z.C. (2014). Influencing factors of mental health of medical students in China. J. Huazhong Univ. Sci. Technol. Med. Sci..

[38] Zheng X., Guo Y., Yang H., Luo L., Ya B., Xu H., Xue Z., Li Q., Shi J., Bi J. (2021). A Cross-Sectional Study on Mental Health Problems of Medical and Nonmedical Students in Shandong During the COVID-19 Epidemic Recovery Period. Front. Psychiatry.

[39] Lee J., Jeong H.J., Kim S. (2021). Stress, Anxiety, and Depression Among Undergraduate Students during the COVID-19 Pandemic and their Use of Mental Health Services. Innov. High Educ..

[40] Zhao L., Sznajder K., Cheng D., Wang S., Cui C., Yang X. (2021). Coping Styles for Mediating the Effect of Resilience on Depression Among Medical Students in Web-Based Classes During the COVID-19 Pandemic: Cross-sectional Questionnaire Study. J. Med. Internet Res..

[41] Zhang Y., Liu B. (2021). Psychological Distress Among Chinese College Students During the COVID-19 Pandemic: Does Attitude Toward Online Courses Matter?. Front. Psychol..

[42] Peng X., Liu L., Liang S., Chen J., Zhao J. (2022). Longitudinal changes in fear and anxiety among Chinese college students during the COVID-19 pandemic: A one-year follow-up study. Curr. Psychol..

[43] Kessler R.C., Andrews G., Colpe L.J., Hiripi E., Mroczek D.K., Normand S.L.T., Walters E.E., Zaslavsky A.M. (2002). Short screening scales to monitor population prevalences and trends in non-specific psychological distress. Psychol. Med..

[44] Zhou C., Chu J., Wang T., Peng Q., He J., Zheng W., Liu D., Wang X., Ma H., Xu L. (2008). Reliability and validity of 10-item Kessler scale (K10) Chinese version in evaluation of mental health status of Chinese population. Chin. J. Clin. Psychol..

[45] Frisco M.L., Houle J.N., Martin M.A. (2010). The image in the mirror and the number on the scale: Weight, weight perceptions, and adolescent depressive symptoms. J. Health Soc. Behav..

[46] Hill P.L., Turiano N.A., Mroczek D.K., Roberts B.W. (2012). Examining Concurrent and Longitudinal Relations Between Personality Traits and Social Well-being in Adulthood. Soc. Psychol. Personal. Sci..

[47] Wu X., Ye Y. (2018). Technical and Vocational Education in China.

[48] White H. (1980). A heteroskedasticity-consistent covariance matrix estimator and a direct test for heteroskedasticity. Econometrica.

[49] Ministry of Education of China (2019). Educational Statistics Yearbook of China (2018).

[50] Kessler R.C., Berglund P., Demler O., Jin R., Merikangas K.R., Walters E.E. (2005). Lifetime Prevalence and Age-of-Onset Distributions of DSM-IV Disorders in the National Comorbidity Survey Replication. Arch. Gen. Psychiatry.

[51] Mojtabai R., Olfson M., Han B. (2016). National Trends in the Prevalence and Treatment of Depression in Adolescents and Young Adults. Pediatrics.

[52] Duffy M.E., Twenge J.M., Joiner T.E. (2019). Trends in Mood and Anxiety Symptoms and Suicide-Related Outcomes Among U.S. Undergraduates, 2007–2018: Evidence From Two National Surveys. J. Adolesc. Health.

[53] Knapstad M., Sivertsen B., Knudsen A.K., Smith O.R.F., Aarø L.E., Lønning K.J., Skogen J.C. (2021). Trends in self-reported psychological distress among college and university students from 2010 to 2018. Psychol. Med..

[54] Pedrelli P., Nyer M., Yeung A., Zulauf C., Wilens T. (2015). College Students: Mental Health Problems and Treatment Considerations. Acad. Psychiatry.

[55] Zivin K., Eisenberg D., Gollust S.E., Golberstein E. (2009). Persistence of mental health problems and needs in a college student population. J. Affect. Disord..

[56] Ahmad W.N.W., Idrus A.A., Azman M.N.A., Kassymova G.K. (2022). Correlates of mental health on online distance learning during COVID-19 among Malaysia vocational students. Int. J. Public Health Sci. (IJPHS).

[57] Tesar M. (2020). Towards a Post-COVID-19 ‘New Normality?’: Physical and Social Distancing, the Move to Online and Higher Education. Policy Futures Educ..

[58] Xu D., Jaggars S.S. (2014). Performance Gaps Between Online and Face-to-Face Courses: Differences Across Types of Students and Academic Subject Areas. J. High. Educ..

[59] Mokhtari M., Dehghan S.F., Asghari M., Ghasembaklo U., Mohamadyari G., Azadmanesh S.A., Akbari E. (2013). Epidemiology of mental health problems in female students: A questionnaire survey. J. Epidemiol. Glob. Health.

[60] Dhar B.K., Ayittey F.K., Sarkar S.M. (2020). Impact of COVID-19 on Psychology among the University Students. Glob. Chall..

[61] Saraswathi I., Saikarthik J., Kumar K.S., Srinivasan K.M., Ardhanaari M., Gunapriya R. (2020). Impact of COVID-19 outbreak on the mental health status of undergraduate medical students in a COVID-19 treating medical college: A prospective longitudinal study. PeerJ.

[62] Lopez Steinmetz L.C., Godoy J.C., Fong S.B. (2021). A longitudinal study on depression and anxiety in college students during the first 106-days of the lengthy Argentinean quarantine for the COVID-19 pandemic. J. Ment. Health.

[63] Li H.Y., Cao H., Leung D.Y.P., Mak Y.W. (2020). The Psychological Impacts of a COVID-19 Outbreak on College Students in China: A Longitudinal Study. Int. J. Environ. Res. Public Health.

[64] Fried E.I., Papanikolaou F., Epskamp S. (2022). Mental Health and Social Contact During the COVID-19 Pandemic: An Ecological Momentary Assessment Study. Clin. Psychol. Sci..

[65] Zhang Y., Zhang H., Ma X., Di Q. (2020). Mental Health Problems during the COVID-19 Pandemics and the Mitigation Effects of Exercise: A Longitudinal Study of College Students in China. Int. J. Environ. Res. Public Health.

